# Creativity and Leadership in the Creative Industry: A Study From the Perspective of Social Norms

**DOI:** 10.3389/fpsyg.2021.651817

**Published:** 2021-04-06

**Authors:** Xiaomin Du, Hong Zhang, Shiying Zhang, Ao Zhang, Beibei Chen

**Affiliations:** ^1^School of Economics and Management, Yingkou Institute of Technology, Yingkou, China; ^2^School of Business, Dalian University of Technology, Dalian, China; ^3^Business and Economic Research, Harbin University of Commerce, Harbin, China; ^4^School of Accounting, Jilin University of Finance and Economics, Changchun, China

**Keywords:** individual creativity, social norms, leadership, creative industry, mediating effect

## Abstract

Individual creativity has been the focus of long-term research in creative industries. However, few studies have explored the impact on individual creativity from social factors. At the same time, the influence of individual creativity on the existence of subsequent factors in the creative industry is also worthy of further investigation. From a social standpoint, this research aims to explore how social norms affect individual creativity, and how individual creativity affects subsequent leadership. The present research takes creative entrepreneurs in creative enterprises as the research objects, and the structural equation model is used to analyze the data obtained from 202 valid questionnaires. Besides, the mediating effect of individual creativity between social norms and individual leadership is verified. The results show that social norms can effectively promote the generation of individual creativity that has a positive impact on both transactional or transformational leadership. It is revealed that social norms are effective tools for enhancing creativity, answering the question of how creative ideas are transformed into creative work and leadership. Individual creativity plays a mediating role between social norms and individual leadership.

## Introduction

The creative industry is an important driver of global economic development and knowledge economy, which brings huge economic benefits and job opportunities in innovation and cultural development. With strong ambiguity and uncertainty, startups in the creative industry rely more on artistry, uniqueness, and originality (Chen et al., 2018). Therefore, creativity has become an important driving force for the success of creative companies. At the same time, the creative industry puts more emphasis on the importance of entrepreneurs' characteristics and behaviors. In the process of creative entrepreneurship, creative entrepreneurs are not only cultural creators but also entrepreneurs responsible for their own economic and social security. Therefore, research on individual creativity of creative entrepreneurs has become the focus of scholars' attention, from which the understanding of the relationship between creative ideas, creativity, and entrepreneurship can be enhanced.

As a kind of implicit rule compliance, social norms subtly influence the evaluation of creativity and leadership of social individuals. For emerging industries such as creative industries, the informal system of social norms has a stronger impact on creativity (York and Lenox, 2014). Previous studies have defined creativity as a process by which a person produces novel and practical ideas, focusing on the influence of intrinsic characteristics of the subject of creative ideas on creativity, such as a person's personality, knowledge storage, thinking style, motivation, etc. (Kandler et al., 2016). However, few studies have explored the influence of individual creativity from the perspective of social norms, ignoring the fact that creativity is also a social process subject to social cognition and judgment (Shalley, 2003). At the same time, the empirical research on social norms on creativity is relatively limited, and the research results are somewhat controversial.

In addition, as a start-up of a creative enterprise, how individual creativity will affect leadership, and whether creative work can be effectively transformed into creative leadership also deserves further exploration. In the process of creative entrepreneurship, in addition to using creativity to build a business, creative entrepreneurs must also maintain a balance between creative ideas, creativity, and entrepreneurship, which is achieved through the management and leadership behavior of creative entrepreneurs. In this process, a high level of creativity will give leadership new characteristics (Vessey et al., 2014). Creative entrepreneurs also emphasize the use of their own creativity factors (such as creative cognition, creative thinking, etc.) to identify and analyze external opportunities, make rapid decisions, and ensure the smooth operation of creative entrepreneurship (Chen et al., 2015). At present, the research on creativity and leadership mostly starts from the team and organization level to explore the influence mechanism of leadership on creativity (Shin and Zhou, 2003; Zhang et al., 2011; Wang et al., 2014; Huang et al., 2016). The impact of individual creativity on leadership is relatively rare. Although some literature also reflects some creative leadership behaviors, the relationship between individual entrepreneurial creativity and leadership is still unclear.

Based on the current research status, this research will focus on answering the following questions. First, how do different social norms affect individual creativity? Second, will there be differences in the impact of social norms on individual creativity at two different levels, societal and personal? Third, in creative companies, how does individual creativity affect leadership, and will creativity become a source of leadership? Finally, does individual creativity play a mediating role between social norms and individual leadership? In order to solve the problems above, the present research explores whether there is a difference between different social norms on the creative entrepreneurs' individual creativity. At the same time, this research refines social norms into two levels: societal and personal. From the perspective of the individual self and the social self, it aims to analyze the mechanism of different levels of social norms on individual creativity. Besides, in order to answer whether creativity will become a source of leadership, and thus further solve the problem of how creative ideas are transformed into creative management and leadership, this study draws on the leadership behavior theory and explores the individual creativity of creative entrepreneurs. Finally, the key intermediary role played by individual creativity is explored in the relationship between social norms and individual leadership. Through this research, the question of how creative behavior in the creative industry is accepted and recognized by the social norms is answered. From the perspective of social dynamics, it has deepened the understanding of the activation and driving role of social norms on individual creativity. Moreover, by exploring the impact of individual creativity between social norms and leadership, the present study will help to further understand the new characteristics of creativity given to leadership, and reveal how individual creativity can transform creative work into creative leadership, which will provide a new perspective for leadership research in the creative industry.

## Literature Review

### Social Norms

Social norms are behaviors that a person is expected to follow and expect others to follow in a given social environment (Lapinski and Rimal, 2005; Mcdonald and Crandall, 2015). According to the social norm approach, relevant norms can be divided into descriptive norms and injunctive norms. Descriptive norms are related to the universality of a certain behavior, whose information provided is particularly relevant to interpersonal goals of being effective or accurate (that is, making correct choices); Injunctive norms is related to the degree of social recognition of behavior, establishment, and maintenance, whose interpersonal goals of social relations are particularly relevant (Cialdini et al., 1995). At the same time, different individuals' understanding that exist at the personal and societal level may be varied. Therefore, this research divides descriptive norms and injunctive norms into the personal level and societal level, namely personal descriptive norms, societal descriptive norms, personal injunctive norms, and societal injunctive norms.

### Individual Creativity

Creativity refers to the idea that creative people produce novel and useful ideas at the same time. Specifically, the creativity of creative entrepreneurs reflects the degree of personal talent and intelligence of the founders of the company. The generated ideas are aimed at solving corresponding management problems and obtaining sustainable competitive advantages (Chen et al., 2015). Existing literature believes that creativity, which has a positive impact on the generation of creative ideas, is considered as the starting point of innovation (Baron and Tang, 2011). The creative process is a collection of cognitive and behavioral steps, designing problem identification, extensively searching for various information, looking for new methods, generating ideas from multiple sources, generating solutions, and elaborating development ideas.

### Social Norms and Individual Creativity

Descriptive norms can easily guide the formation of creative behavior (Rivis and Sheeran, 2003). Creative entrepreneurs aim to use their own creativity to generate new and useful ideas. When individuals lack sufficient creative information or information is vague and difficult to make decisions, they will be unconsciously affected by the behavior of others. The behavioral information of the public will form a kind of behavioral norms and social pressure, making it an important situational factor to be considered when forming decisions and judgments, and promoting individual behaviors (Blay et al., 2016). When it comes from important stakeholders, such as the relevant creative behaviors of competitors in the creative industry. While bringing pressure and threats to creative entrepreneurs, it also brings related behavioral model references.

Besides, creativity is a social construct and consensus attribution. The dual process of performing creativity and being recognized is the game process between creative entrepreneurs and consumers. In the early stage of creative processes, creative entrepreneurs search and identify the information and opportunities from the outside world to serve as a source of creativity (Lapinski et al., 2017). Therefore, the universality of a certain social behavior will become the basis of creativity. Meanwhile, creative entrepreneurs have social identities, which derived from the membership of social groups, including (internalized) values and norms. The distinctiveness of social identity prompts people to follow group norms and influences the creative processes. When individuals face the threat of social group identity, they are more likely to abide by the descriptive norms (Hirst et al., 2009; Grant and Berry, 2011). Observing the social descriptive norms can show others their own identification with the group they belong to, so as to reduce the individual's anxiety and discomfort (Liu et al., 2019). Therefore, descriptive norms promote individual creative motivation and affect individual creative behavior.

Hypothesis 1: Personal descriptive norms positively affect individual creativity.
Hypothesis 2: Societal descriptive norms positively affect individual creativity.

Injunctive norms are the value judgments of most group members. Personal injunctive norms will bring certain pressure and threats to individuals (Fugas et al., 2011). Under emergencies and threats, individuals have an incentive to explore novel solutions to solve pressing problems. Amabile et al. (1996) believes that challenging stress perception and load stress perception are important work environment perceptions that affect an individual's level of creativity (Sacramento et al., 2013). Challenging pressure perception can enhance employees' internal motivation and have a positive effect on individual creativity. The injunctive norms at the individual level will inspire an individual's systematic information processing mode including divergent and aggregated thinking. Divergent and aggregated thinking modes are the prerequisites for constructing creative ideas.

Social sanctions are used as guarantees for behavioral execution, and people's behaviors are guided by emphasizing the behaviors (Yuan and Wu, 2020). When individuals' attitudes and behaviors are supported by social injunctive norms, creative entrepreneurs tend to act according to their original attitudes (Gelfand et al., 2017; Yuan et al., 2020). When creative behavior is not recognized by injunctive norms, creative entrepreneurs will use creativity to adjust creative behavior. Social trust, confidence, and praise for individuals will increase their belief in creative entrepreneurship, and thus enhance their sense of effectiveness, which will help increase individual creativity.

Hypothesis 3: Personal injunctive norms positively affect individual creativity.
Hypothesis 4: Societal injunctive norms positively affect individual creativity.

### Leadership

According to the leadership theory, Longshore and Bass (1987) believes that transactional and transformational leadership can be reflected in the same person. Thus, this study aims to explore transactional leadership and transformational leadership. Transactional leadership is defined as the ability to clearly assign tasks, indicate specific divisions of labor, and influence employees through rewards and punishments with clear interests. Transformational leadership refers to a leadership behavior that stimulates the creativity, identity, and risk-taking motivation of subordinates in a complex environment with vision incentives, intellectual inspiration, leadership charm, and compassionate care (Bass, 1990; Makri and Scandura, 2010).

The characteristics of creative industries determine that the specific functions of creative leadership are different from the usual leadership. Creative work is closely related to the planning of creative leadership for long-term goals, the guidance of creative employees, the construction of organizational culture, and so on. One of the key goals of leadership in the creative industries is to motivate creative people to produce both profitable and creative work. Therefore, leadership in the creative field lies in how to make creative people give full play to their working skills and stimulate their creativity to the maximum extent.

### Individual Creativity and Leadership

During creative processes, three different phases are designed, including identification of business opportunities, acquisition of resources, business profitability and growth (Baron and Tang, 2011; Gielnik et al., 2012). Most companies in the creative industry are new ventures, and there are no established business models or key success factors before. Therefore, entrepreneurs will send trading signals to the future prospects for human resources such as education, industry experience, and entrepreneurial experience in order to obtain external resources and investments (Ko and Mckelvie, 2018). When startups can prove that they have the potential to provide a considerable return on investment, they can use external resources for further development.

Creative industry has certain ambiguities and uncertainties, which pose great challenges to creative entrepreneurs and creative workers. When employees can accomplish the development goals of the organization well, valuable resource rewards will be provided for employees. When the employee's behavior is wrong or does not meet the standard, the leader will give feedback, make corrections, or impose punishment.

Hypothesis 5: Individual creativity will positively affect transactional leadership.

Leaders with a high level of creativity are good at using creative thinking for meaning construction, leading cross-border members to discover state-of-the-art technologies, new markets, and novel knowledge. Besides, efficient creative operation mechanisms will be established then. At the same time, leaders will continue to encourage members to adapt to environmental changes, use cooperative innovation to find opportunities, reduce members' anxiety about complex environments, and turn them into development momentum. The creation of ideas is often accompanied by contradictions and conflicts (Miron-Spektor et al., 2011). Creative entrepreneurs need to actively deal with conflicts, rather than blindly suppress the tensions existing in the organization (Jørn and Schei, 2010; Chen at al., 2015). By openly and critically handling conflicts in an organization, the cognitive behavior and behavioral complexity of creative entrepreneurs can be changed, and the creative insights that drive organizational change might be produced.

Hypothesis 6: Individual creativity will positively affect transformational leadership.

### The Mediating Role of Individual Creativity

Social norms can be seen as a form of social control, which homogenizes the individual's thoughts and behaviors, which can also be tools to enhance creativity and leadership behavior. In creative activities, creative entrepreneurs need to pay attention to two aspects of leadership at the same time: creative worker behavior and creative manager performance. Creative workers aim to propose creative methods to solve problems. Meanwhile, the creativity of leaders in the management process has an important influence on leadership. For example, creative leaders are not limited to traditional rules and regulations to restrain their subordinates, and they are more open to the creative activities of employees, which will help them to exert their leadership.

**Figure 1:**
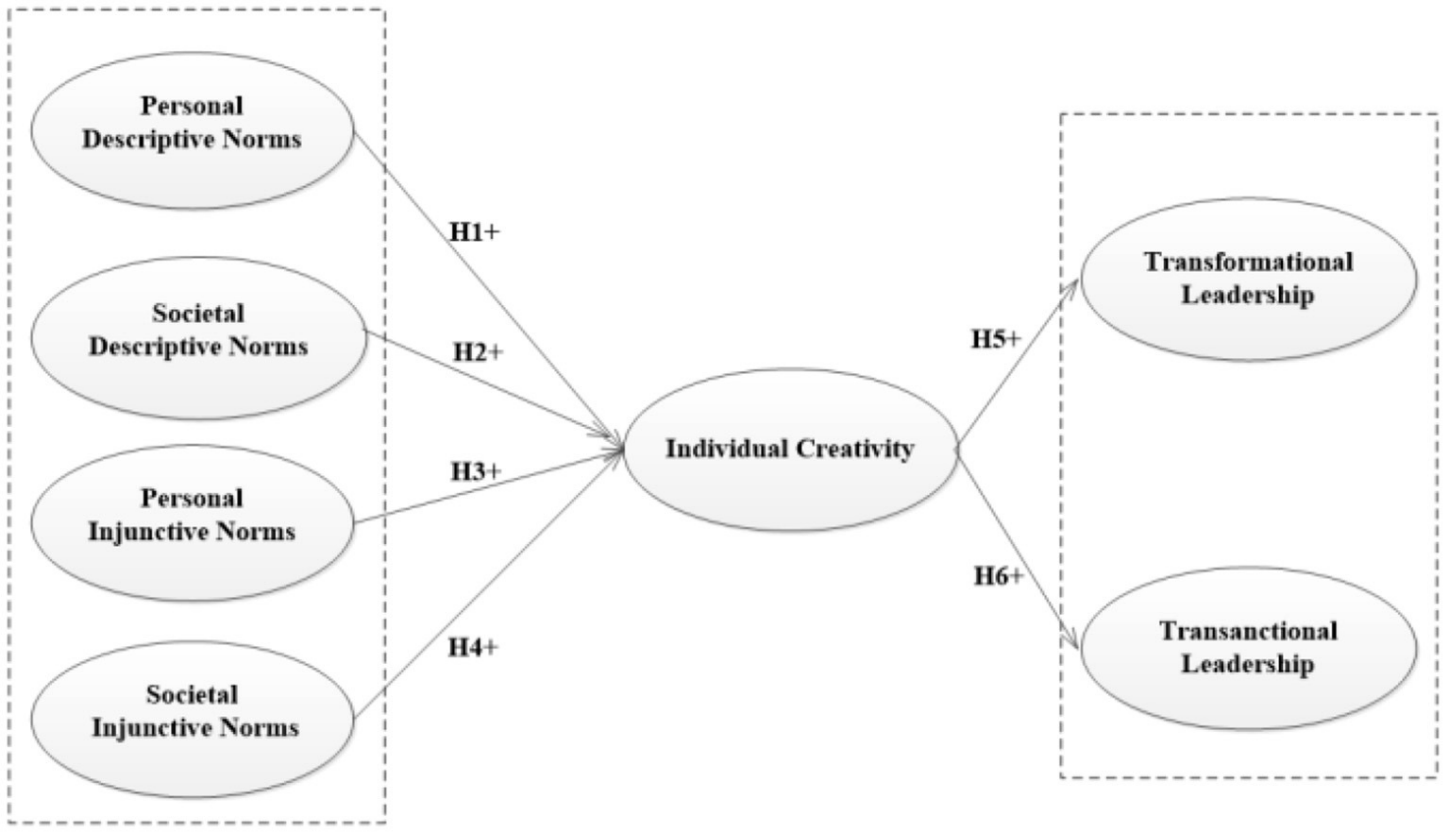
Conceptual framework.

Hypothesis 7: Individual creativity plays an intermediary role in the relationship between social norms and individual leadership. The conceptual framework is shown in Figure 1.

## Method

### Research Design and Data Collection

The present study adopted the definition of the creative industry proposed by the British Ministry of Culture, Media, and Sports. The process of collecting data is as follows. First, in order to ensure the representativeness, we collected 100 cases (a total of 300 companies) from the three regions of eastern China, central China, and western economic zone. Their contact information can be obtained through Tianyancha- an enterprise information query platform. Second, we contact the main founder of each company such as CEO, general managers, or owners via different channels. Before the survey began, we specified the purpose of the survey, and guaranteed that the survey data will only be used for academic research. Finally, after confirming the contact information of the participants, we issued the corresponding questionnaire. We use the questionnaire collection tool to send the questionnaire's link to entrepreneurs. From June 2019 to January 2020, the data collection process took 7 months and gathered 274 questionnaires in total. However, in order to ensure the validity of the questionnaires' results, 62 invalid questionnaires were deleted. Finally, we got 202 valid questionnaires. The statistics of the samples are shown in Table 1.

**Table 1:** Characteristics of the research samples (*N* = 202).

**Control variables**	**Item**	**Frequency**	**Percentage**
Gender	Male	109	54.0
	Female	93	46.0
Age	≤ 30	69	34.2
	32–35	65	32.2
	36–40	25	12.4
	41–45	13	6.4
	≥46	30	14.9
Educational background	High School	22	10.9
	Bachelor	84	41.6
	Master	53	26.2
	Doctor	15	7.4
	Others	28	13.9
Industry	Advertising	35	17.3
	Design	19	9.4
	Software	58	28.7
	Filming and TV	21	10.4
	Music and Picture	18	8.9
	Art and Publishing	15	7.4
Year of establishment	≤ 1	15	7.4
	1–3	44	21.8
	3–5	29	14.4
	5–10	30	14.9
	≥10	84	41.6

### Measures

#### Explanatory Variables: Social Norms

Social norms use direct measures of variables in the planned behavior theory (TPB) to allow parallel measurement of different social norms (Park and Smith, 2010). Among them, the Cronbach's α coefficients of the different social norms are respectively 0.882, 0.876, 0.921, and 0.970.

#### Mediating Variable: Individual Creativity

Entrepreneur's individual creativity is measured as a set of personality characteristics (Amabile et al., 1996). This research uses creativity scale developed by Amabile to measure individual creativity. Its Cronbach's α coefficient is 0.967.

#### Explained Variable: Leadership

To measure transformational leadership and transactional leadership, this study applied a multi-factor leadership competency questionnaire to measure them (Bass, 1990). Among them, transactional leadership has a total of 12 items, and transformative leadership has six items. The Cronbach's α coefficient for transactional leadership is 0.968, and the Cronbach's α coefficient for transformational leadership is 0.951.

All measurements are measured using Likert's 5-level scale. Among them, “1” means “strongly disagree,” and “5” means “strongly agree.” In the empirical analysis of the present study, gender, age, industry, education level, and age were employed as control variables.

### Reliability and Validity

#### Reliability

In order to verify the validity of the questionnaire and the data in this survey, the present study uses AMOS software to test the reliability and validity of the collected data. To test the reliability of the scale, Cronbach's α and construction factor loads were used. The results are shown in Table 2. In terms of subject reliability, the standardized coefficients of each subject are >0.6 and reach a significant level, and the SMC value is >0.36, which achieves the ideal subject reliability level. The composition reliability (CR) is >0.8, which achieves the ideal composition reliability value, proving that the model has good reliability.

**Table 2:** Validity and reliability of construct measures (*N* = 202).

**Construct**	**Item**	**Std**	**SMC**	**AVE**	**CR**
Personal descriptive norm	PD1	0.861	0.741	0.715	0.882
	PD2	0.906	0.821		
	PD3	0.763	0.582		
Societal descriptive norm	SD1	0.827	0.684	0.641	0.876
	SD2	0.852	0.726		
	SD3	0.840	0.706		
	SD4	0.670	0.449		
Personalinjunctive norm	PI1	0.893	0.797	0.795	0.921
	PI2	0.912	0.832		
	PI3	0.870	0.757		
Societalinjunctive norm	SI1	0.805	0.648	0.683	0.970
	SI2	0.929	0.863		
	SI3	0.909	0.826		
	SI4	0.847	0.717		
Individual creativity	IC1	0.686	0.471	0.661	0.967
	IC2	0.870	0.757		
	IC3	0.885	0.783		
	IC4	0.839	0.704		
	IC5	0.803	0.645		
Transactional leadership	TL1	0.785	0.616	0.668	0.968
	TL2	0.779	0.607		
	TL3	0.811	0.658		
	TL4	0.813	0.661		
	TL5	0.803	0.645		
	TL6	0.800	0.641		
	TL7	0.821	0.674		
	TL8	0.850	0.723		
	TL9	0.785	0.616		
	TL10	0.845	0.714		
	TL11	0.828	0.686		
	TL12	0.724	0.524		
Transformational leadership	RL1	0.859	0.738	0.765	0.951
	RL2	0.891	0.794		
	RL3	0.853	0.728		
	RL4	0.872	0.760		
	RL5	0.912	0.832		
	RL6	0.861	0.741		

#### Validity

AMOS is used to test the aggregation validity and differentiation validity of the questionnaire. The results show that the factor load exceeds 0.7, and the average variance extraction (AVE) value reflects the average explanatory ability of each topic for all problems of the facet. Theoretically, it is recommended to be >0.5. The AVE of this study meets the standard, proving that the model has good convergence validity.

In this study, AVE method was used to evaluate the discrimination validity. According to the recommendations of Fornell and Lacker, the square root value of AVE for each facet must be greater than the correlation coefficient of each pair of variables, indicating that the facets have distinguishing validity. The results of this study are shown in Table 3. The statistics in bold type are the square root values of AVE in each dimension, and they are greater than the normalized correlation coefficients below them, so this study has good discrimination validity.

**Table 3:** Correlation analysis.

	**Convergence validity**	**Discrimination validity**						
	**AVE**	**1**	**2**	**3**	**4**	**5**	**6**	**7**
Transactional leadership	0.668	**0.817**						
Transformational leadership	0.765	0.669	**0.875**					
Individual creativity	0.661	0.694	0.802	**0.813**				
Societal injunctive norms	0.683	0.547	0.64	0.744	**0.826**			
Personal injunctive norms	0.795	0.652	0.709	0.824	0.739	**0.892**		
Societal descriptive norms	0.641	0.732	0.716	0.826	0.618	0.824	**0.801**	
Personal descriptive norms	0.715	0.618	0.477	0.558	0.453	0.543	0.536	**0.846**

*The statistics in bold type are the square root values of AVE, and the data below them are the Pearson correlations of the dimension*.

## Results

The fitting results of the structural equation model are as follows: χ^2^ = 1469.106, df = 589, χ^2^/df = 2.494, SRMR = 0.0673, CFI = 0.900, and RMSEA = 0.08, which reached the acceptable fitting standard.

**Figure 2:**
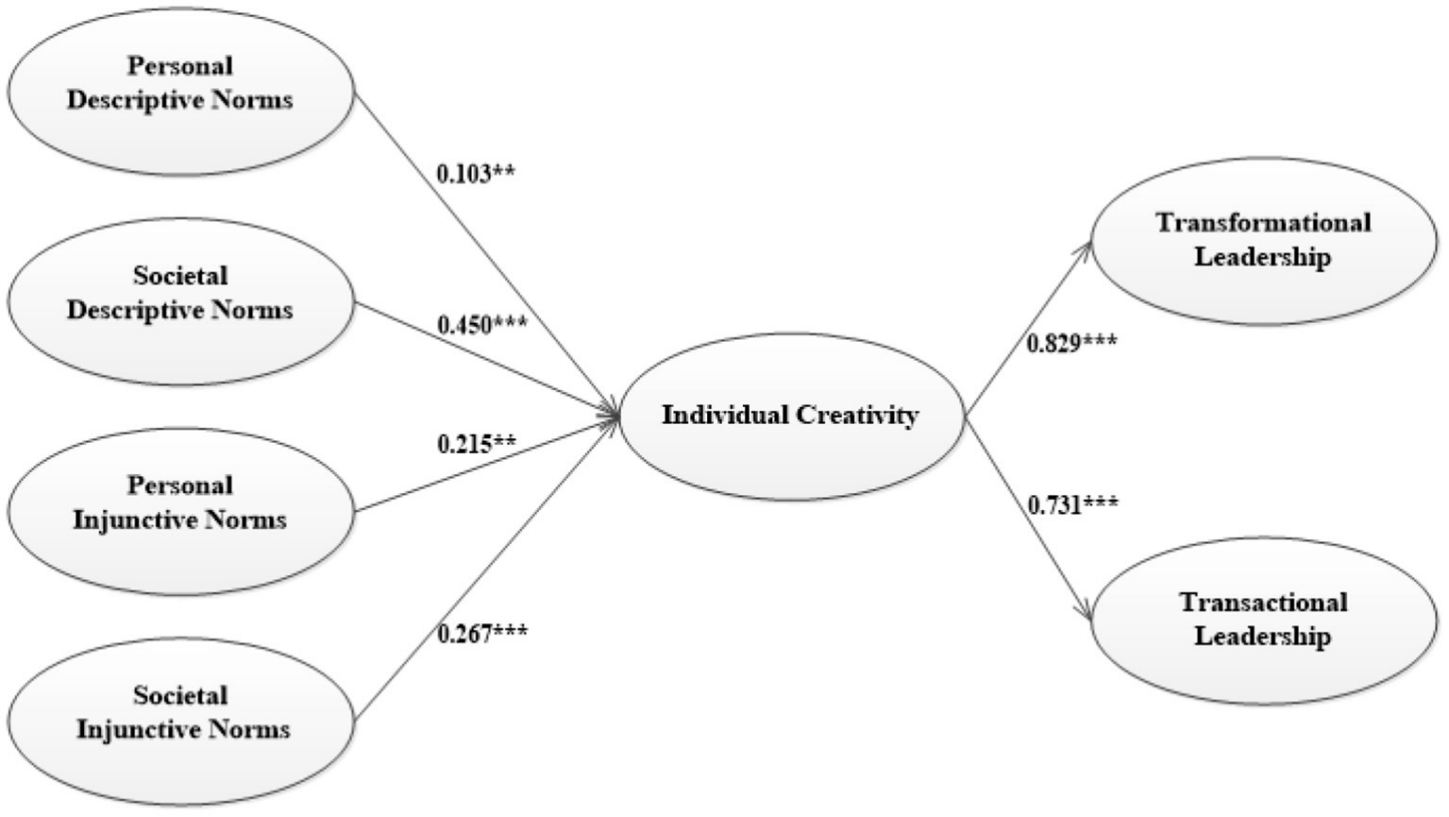
Results of structural equation modeling. ^***^p < 0.001; ^**^p < 0.05.

Table 4 and Figure 2 display the path coefficients of the model. Hypotheses 1 and 2 are that descriptive norms' impact on individual creativity at personal or societal level. It can be drawn from Table 4 that personal descriptive norms have a significant positive effect on individual creativity (β = 0.103, *p* < 0.05). Societal descriptive norms have a significant positive effect on individual creativity (β = 0.450, *p* < 0.001). Thus, Hypotheses 1 and 2 are accepted. When it comes to societal injunctive norms, the relationship between societal injunctive norms and individual creativity is significant (β = 0.267, *p* < 0.001). Personal injunctive norms have a significant positive effect on individual creativity (β = 0.215, *p* < 0.05). Both societal and personal injunctive norms have positive effects on individual creativity. Thus, Hypotheses 3 and 4 can be accepted. At the same time, *R*^2^, which represents the independent variable's ability to explain the dependent variable (individual creativity), is 0.832, meeting the standard of credibility, which can well prove the first four hypotheses of this study. For Hypothesis 5 and Hypothesis 6, the present study proposes that individual creativity will be positively related to transactional leadership and transformational leadership. The relationship between individual creativity and transactional leadership is significantly positive (β = 0.731, *p* < 0.001), which means individual creativity has an effect on transactional leadership. The relationship between individual creativity and transformational leadership is significantly positive (β = 0.829, *p* < 0.001), which is consistent with our hypothetical predictions. The ability of independent variable (creativity) to explain two dependent variables (transactional/transformational leadership) is 0.535 and 0.687, which has reached the ideal level, indicating that each dependent variable can be well-explained.

**Table 4:** Hypothesis testing results.

**DV**	**IV**	**Unstd**.	**S.E**.	**C.R**.	***P***	**Std**.	***R*^**2**^**
Individual creativity	Personal descriptive norms	0.084	0.043	1.969	0.049	0.103	0.832
	Societal descriptive norms	0.381	0.091	4.17	0.000	0.450	
	Personal injunctive norms	0.165	0.083	1.987	0.047	0.215	
	Societal Injunctive norms	0.246	0.062	3.955	0.000	0.267	
Transactional leadership	Individual creativity	0.752	0.090	8.372	0.000	0.731	0.535
Transformational leadership	Individual creativity	0.953	0.098	9.706	0.000	0.829	0.687

For Hypothesis 7, the Bootstrap method (the bias-corrected percentile method and the percentile method) was further applied to test the mediating effect of personal creativity on the relationship between social norms and leadership. Bootstrap was set to draw 1,000 samples, and the confidence interval level was 95%. If the upper limit and lower limit of the confidence interval do not contain 0, the point estimate will be significant.

As shown in Table 5, the indirect effects, direct effects, and total effects in the model are all significant, indicating that there are indeed some mediating effects. Social norms have a significant impact on personal creativity, and the standardized influence coefficient is 0.81 (*P* < 0.001). Personal creativity has a significant impact on leadership, and the standardized impact coefficient is 0.48 (*P* < 0.001). Social norms have a significant impact on leadership, and the standardized impact coefficient is 0.47 (*P* < 0.001).

**Table 5:** Bootstrap mediating effect test.

	**Point estimate**	**SE**	**Bootstrap 1,000 times 95% CI**			
			**Bias corrected**		**Percentile**	
			**Lower**	**Upper**	**Lower**	**Upper**
IE	0.393	0.215	0.044	0.955	0.009	0.866
DE	0.470	0.180	0.147	0.842	0.037	0.79

## Conclusion

The present study on individual creativity focuses not only on the influence of internal factors such as personal characteristics and motivation but also on the influence of social factors related to creativity (Grant and Berry, 2011). The purpose is to explore the relationship among social norms, individual creativity, and leadership. From a social perspective, this study reveals how social norms affect the generation of individual creativity, and how individual creativity affects subsequent leadership. Whether individual creativity plays an intermediary role among them is also explored.

According to different types and levels of norms, this study divides social norms into descriptive norms and injunctive norms, both of which are defined on the individual and the societal levels. Results first show that both norms are significant sources of individual creativity, which reflect people's perception of others' recognition or what they should do. This also confirms the previous research that social norms are an important factor affecting social behavior (Goncalo et al., 2014). However, the two norms have slightly different mechanisms for individual creativity. Descriptive norms emphasize the typicality and normality of behavior, providing evidence to explain what is appropriate and effective to stimulate creative behavior; injunctive norms emphasize the influence of creative behavior through potential individual or social rewards and punishments participating in the process of creation. At the personal level, creative entrepreneurs share two perceptions in society, namely self-perception and social perception. The individual's cognition of external norms depends on the self-perception of his/her behavioral characteristics or state (Kunrath et al., 2020). When self-concept is emphasized, creative entrepreneur's creativity is more susceptible to personal goals and needs. Self-perception is the origin of social cognition. From the social perspective, a society and collectives in a certain social network give creative entrepreneurs collective self-awareness and social perception (Long and Wang, 2019). When social perception is emphasized, entrepreneur's creativity is more susceptible to collective norms and values, and they tend to act in accordance with external values, social norms, and expectations. China is dominated by a highly relationship-oriented culture, which attaches great importance to collectivist values and the Confucian culture. Therefore, in the context of Chinese collectivism, creative entrepreneurs place more emphasis on the attributes of the collective self, and the cognition of external norms will come from the self-evaluation of specific groups. The conclusion of this paper well verifies the social identity theory driven by social norms and the theory of norm activation.

Second, the impact of individual creativity on back-end leadership has been verified. Results show that individual creativity of has a positive impact on leadership. The key difference between them is the form of interaction between leaders and subordinates. Transactional leaders often correctly accomplish things, clearly give creativity roles and tasks, and coherently provide incentives and exceptional management through the exchange of value and benefits; however, transformational leadership, which employs one's own creativity to build an environment suitable for creativity, employee encouragement, and the mobilization of a variety of creative incentive mechanisms, ensures the smooth operation of the creation process. Although different kinds of leaderships have different influence mechanisms, creativity can provide a transformation path for both types of leadership. Previous studies have pointed out that transformational leadership and transactional leadership promotes creativity, while this study proves that individual creativity also has a positive effect on leadership (Qu et al., 2015). Therefore, in a creative agency or team, both transformational and transactional leaders need to recognize that creativity is one of the most important leadership factors in organizational efficiency and future success.

The present study produces the following contributions. First, from the perspective of social norms, it is possible to fundamentally reveal the social attributes to individual creativity and leadership, which may enhance scholars' understanding of the social drive of individual creativity and leadership. With the continuous development of information technology and social network, the social network with network as the medium arises at the historic moment. Information and events in the society will provide creative sources for creative entrepreneurs. Creative entrepreneurs can implement their ideas by exerting their creativity and leadership, and produce corresponding creative products to meet the ever-developing personalized and diversified market demands. Second, this study further demonstrates the relationship between theoretical creative ideas, creativity, and entrepreneurship. Social events can be a source of creative ideas, and social norms can influence the direction in which ideas form creative products. According to this direction, creative entrepreneurs use their creativity to produce creative ideas into formative creative products that can enter the market. Creative products that conform to social norms and meet the creative needs of social groups will become the sustainable competitiveness of creative entrepreneurship (Wu et al., 2018). Creative entrepreneurs influence creative management and leadership through creative ideas to form a creative entrepreneurial environment, ensuring the smooth progress of the creative process. The present study provides a new perspective on the behavioral theory of leadership and outlines a strategic framework that affects individual creativity and leadership. Moreover, creativity promotes leadership. In the past, creativity was only applied as an optional factor for leadership construction, but now it is essential in the enterprise. Therefore, it is essential to explore the promoting role of creativity in the process of creative management and leadership to produce competitive creative products. Moreover, individual creativity plays an intermediary role between social norms and individual creativity. This study initially reveals how creative ideas are transformed into creative management and creative leadership.

This paper has some limitations, but also provides a direction for future research. First, the research object of this paper is the creativity and leadership of creative entrepreneurs affected by social norms in creative industries. The generation and execution of creative ideas are driven by individual mental activities of creative entrepreneurs, and creative capital is formed through creativity and leadership. At the same time, there is a process of value co-creation between creative industries and social groups, so social norms have certain particularity to the creativity and leadership of creative industries. However, whether the conclusions of this study can be applied to other industries and industries remains to be further studied. Second, this study was conducted in the context of China's creative industry, which is influenced by Confucian culture, where individuals tend to follow the behavior of the masses. Social norms may have different influences on creativity and leadership in different cultural contexts. Therefore, future research can start from the background of social norms in different countries to explore the social factors that enhance creativity and leadership. Third, the research method of questionnaire survey is adopted in this study. Different subjects have different understandings of the same item, then the questionnaire results may have certain influence on the final empirical results. The questions used in the questionnaire are the ones in the mature scale, which may omit some more detailed and deep information. Therefore, the following research can use the method of case study to explore the influence path of social norms on creativity and leadership, and conduct an in-depth analysis of the social factors of creativity and leadership.

## Data Availability Statement

The raw data supporting the conclusions of this article will be made available by the authors, without undue reservation.

## Ethics Statement

Ethical review and approval was not required for the study on human participants in accordance with the local legislation and institutional requirements. The patients/participants provided their written informed consent to participate in this study. Written informed consent was obtained from the individual(s) for the publication of any potentially identifiable images or data included in this article.

## Author Contributions

XD and HZ: writing. SZ: providing idea. AZ and BC: providing revised advice. All authors contributed to the article and approved the submitted version.

## Conflict of Interest

The authors declare that the research was conducted in the absence of any commercial or financial relationships that could be construed as a potential conflict of interest.
